# Spatial Porosity as a Diagnostic Predictor of Conductivity Collapse in Patient-Specific Radiofrequency Ablation of Liver Tumors

**DOI:** 10.3390/diagnostics16111610

**Published:** 2026-05-25

**Authors:** Nikola Bošković, Branislav Radjenović, Štefan Matejčik, Marija Radmilović-Radjenović

**Affiliations:** 1 Institute of Physics, University of Belgrade, Pregrevica 118, 11080 Belgrade, Serbia; nikolab@ipb.ac.rs (N.B.); bradjeno@ipb.ac.rs (B.R.); 2 Faculty of Mathematics, Physics, and Informatics, Comenius University, Mlynskadolina F2, 84248 Bratislava, Slovakia; stefan.matejcik@fmph.uniba.sk

**Keywords:** radiofrequency ablation, multiphysics modeling, tissue heterogeneity, diagnostic impedance monitoring, porosity mapping, interventional oncology

## Abstract

**Background**: Radiofrequency ablation of liver tumors relies on tightly coupled electromagnetic–thermal dynamics. However, conventional computational models oversimplify tissue heterogeneity and the dynamic evolution of biophysical properties, limiting their intraoperative diagnostic utility. **Methods**: We developed a patient-specific, three-dimensional multiphysics framework for liver RFA that integrates spatially varying tissue porosity with a modified local thermal equilibrium formulation. Advective heat transfer is computed via a supplementary finite-element equation, fully coupled with quasi-static electromagnetic simulations and Arrhenius-based tissue damage kinetics. **Results**: Simulations revealed three distinct voltage-dependent regimes: stable thermal–electromagnetic coupling at 50 V, optimal lesion expansion at 75 V, and premature electrical conductivity collapse at 100 V. Dynamic conductivity reduction, driven by dehydration and coagulative necrosis, provides a mechanistic basis for interpreting real-time impedance rises as an early indicator of peri-electrode desiccation. Geometry-constrained porosity mapping accurately reproduced anisotropic lesion morphologies, yielding simulated necrotic diameters of 2.8 ± 0.4 cm, closely aligning with MRI-validated clinical benchmarks. **Conclusions**: By linking microstructural heterogeneity to electromagnetic feedback, this framework transforms intraoperative impedance monitoring into a quantitative, predictive diagnostic tool. Imaging-derived spatial porosity mapping represents a robust biomarker for patient-specific liver RFA planning, significantly reducing procedural uncertainty and improving ablation precision.

## 1. Introduction

Radiofrequency ablation is a minimally invasive thermal therapy widely employed for the local treatment of primary and metastatic solid tumors [1,2,3,4,5,6]. The technique relies on delivering alternating electrical current through an inserted electrode, inducing resistive (Joule) heating that triggers coagulative necrosis [7,8,9,10]. Due to its reduced invasiveness and compatibility with image-guided interventions, RFA has emerged as a cornerstone therapeutic modality for patients unsuitable for surgical resection [11,12,13,14].

The therapeutic efficacy of RFA is governed by the spatiotemporal distribution of deposited electromagnetic (EM) energy and the resulting thermal field, which collectively dictate the extent of tissue necrosis [15,16,17]. In contrast to microwave ablation (MWA), where dielectric heating via EM wave propagation predominates, or cryoablation, which relies on freeze–thaw cycles to induce cellular injury through ice-crystal formation and vascular stasis, RFA operates in the quasi-static regime. Consequently, local power deposition is predominantly determined by tissue electrical conductivity [18,19,20,21]. Because conductivity exhibits a strong nonlinear dependence on temperature and tissue state that initially increases with heating before collapsing due to dehydration and protein denaturation, accurate prediction of its dynamic evolution is critical for estimating ablation margins, avoiding premature impedance roll-off, and optimizing treatment outcomes.

Computational modeling has emerged as a vital tool for understanding the biophysical mechanisms of thermal ablation and for optimizing patient-specific treatment planning [22,23,24,25,26,27]. Nevertheless, many existing RFA models rely on the assumption of homogeneous tissue properties or employ oversimplified representations of biological heterogeneity [28,29,30]. In reality, tumor and peritumoral microarchitecture exhibits substantial spatial variability, particularly in vascular density, extracellular matrix composition, and interstitial fluid content, which is macroscopically characterized by tissue porosity [31,32,33,34]. Moreover, prior studies frequently adopt axisymmetric or idealized geometries, which inherently constrain the representation of three-dimensional heterogeneity present in real tumors.

During ablation, dehydration, protein coagulation, and carbonization dynamically remodel the tissue microstructure, generating localized zones of markedly reduced electrical conductivity. These regions perturb current pathways and redistribute Joule heating, thereby influencing lesion growth and morphology. Crucially, the bidirectional coupling between evolving tissue properties and electromagnetic energy deposition remains inadequately captured in conventional modeling frameworks, limiting their ability to predict impedance dynamics and ablation margins with clinical fidelity.

Modeling heat transfer in biological tissue poses additional challenges [35,36,37,38,39]. Classical bioheat models based on local thermal equilibrium (LTE) approximate perfusion as an isotropic source term, neglecting directional advective transport. Although modified LTE formulations include advective contributions, the effective velocity field is typically prescribed empirically without rigorous physical justification [31]. To address these limitations, we propose a modified LTE formulation in which advective transport is computed via an auxiliary finite-element equation evaluating the scalar product of an effective radial velocity vector and the temperature gradient. This velocity field represents fluid motion radiating outward from the electrode, consistent with the cylindrical symmetry of current distribution, providing a physically grounded representation of volume-averaged perfusion [40,41].

This study integrates the above thermal model into a patient-specific, three-dimensional multiphysics framework to investigate the coupled electromagnetic–thermal dynamics of RFA, explicitly accounting for structural tissue heterogeneity through spatially varying porosity. Coupled with a quasi-static electromagnetic solver and an Arrhenius tissue damage model for dynamic property updates, the framework simulates conductivity evolution across varying applied voltages and ablation durations. The primary objective is to delineate regions of conductivity collapse driven by peri-electrode desiccation, thereby linking microstructural heterogeneity to macroscopic impedance dynamics and enhancing the quantitative interpretation of image-guided ablation.

## 2. Materials and Methods

### 2.1. Mathematical Model

RFA is modeled as a coupled electromagnetic–thermal process in which electrical energy delivered via an inserted electrode is converted into heat through Joule heating. The computational framework integrates quasi-static electromagnetic conduction, advective heat transfer in porous tissue, and temperature-dependent coagulative necrosis into a unified multiphysics formulation. Macroscopic volume-averaging is employed to capture spatially heterogeneous tissue properties while preserving computational efficiency at clinically relevant scales. At typical RFA operating frequencies (≈500 kHz), displacement currents are negligible compared to conduction currents. Under this quasi-static approximation, the electric potential distribution *V* is governed by the charge conservation equation [31,42]:

(1) $$\nabla \cdot (\sigma \left(T,\Omega ,\varepsilon_{\mathrm{var}}\right)\nabla V)=0.$$

The electric field is derived from the potential gradient as E = −∇V, yielding the volumetric Joule heating source $Q_{\mathrm{RF}}=\sigma {\left|\nabla V\right|}^{2}$ in the bioheat equation. Electrical conductivity σ is a state-dependent property governed by local temperature *T*, accumulated thermal damage Ω, and spatially varying porosity $\varepsilon_{\mathrm{var}}$. Because σ scales directly with tissue water content, it initially increases with moderate heating but undergoes a sharp decline near the vaporization threshold (≈100 °C) due to dehydration and phase change [31,42]:

(2) $$\sigma \left(T\right)=\sigma_{0}\cdot f\left(T\right),$$

where $\sigma_{0}$ is the electrical conductivity at the reference body temperature (37 °C), and f(T) is a piecewise function capturing thermal enhancement and vaporization-induced drop.

**Figure 1:**
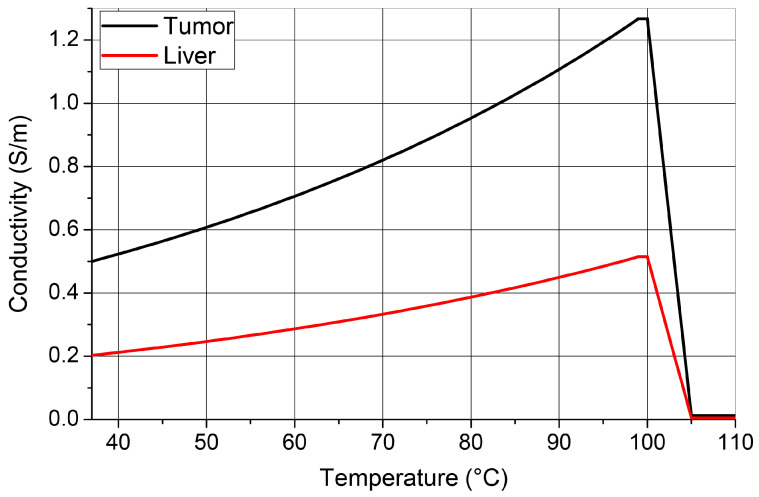
Temperature-dependent electrical conductivity of healthy liver tissue (red) and tumor tissue (black). Nonlinear trends reflect thermal enhancement below 100 °C due to increased ionic mobility, and conductivity collapse above 100 °C driven by dehydration and vaporization [31,42].

As illustrated in Figure 1 (computed using Equation (2) and parameters from Table 1), electrical conductivity differs markedly between healthy liver parenchyma and tumor tissue, with healthy tissue generally exhibiting higher baseline values. Both tissues follow a characteristic nonlinear trend: conductivity increases with temperature up to ≈100 °C due to enhanced ionic mobility, then undergoes a sharp decline driven by dehydration and vaporization, reaching a residual plateau in desiccated, coagulated tissue. Because conductivity directly governs resistive power deposition $Q_{\mathrm{RF}}$, maintaining tissue temperature in the vicinity of 100 °C, while avoiding premature desiccation, it optimizes electromagnetic energy coupling and ablation efficiency.

The tumor tissue properties adopted in this study correspond to hepatocellular carcinoma (HCC), the most prevalent primary liver malignancy treated with RFA [2,6]. While metastatic lesions (e.g., colorectal) exhibit distinct thermophysical characteristics, the present framework is readily adaptable through substitution of tissue-specific parameters from Table 1. Regarding underlying parenchymal disease, the baseline model assumes non-cirrhotic liver tissue; however, the porosity field $\varepsilon_{\mathrm{var}}$ can be calibrated to reflect fibrotic or steatotic conditions by adjusting baseline porosity and perfusion parameters, as outlined in recent porous-media formulations [31,33].

Thermal transport is governed by a modified LTE formulation [31] derived from volume-averaged porous-media theory. Beyond conduction, tissue perfusion is modeled as an advective heat transfer mechanism driven by effective microvascular motion. Unlike classical LTE approaches that approximate perfusion via isotropic volumetric source terms, the present framework explicitly resolves directional heat transport through the convective term $v_{\mathrm{eff}}\cdot \nabla T$, thereby capturing macroscopic advective effects inherent to anisotropic microvascular architecture.

The effective velocity field $v_{\mathrm{eff}}$ is prescribed as a radially outward vector centered on the ablation electrode, aligning with the cylindrical symmetry of the current density and thermal gradients. Rather than resolving discrete vasculature, $v_{\mathrm{eff}}$ represents a homogenized transport mechanism consistent with porous-media averaging, capturing preferential heat dissipation away from the heat source. The advective contribution is evaluated through an auxiliary finite-element equation, replacing empirical perfusion coefficients with a physically consistent closure for the volume-averaged energy balance.

Irreversible tissue damage is quantified using the Arrhenius kinetic model [42,43], with electromagnetic and thermal properties updated dynamically as functions of temperature and accumulated necrosis. Following established protocols [41], the necrotic fraction is defined as $\theta_{d}=1-\exp\left(-\Omega \right)$, where complete coagulation is assumed at Ω≥4.6 (corresponding to $\theta_{d}\geq 0.99$).

### 2.2. Modified Local Thermal Equilibrium Formulation

Heat transfer during radiofrequency ablation involves conductive diffusion, advective transport due to blood perfusion, and thermally induced tissue alterations. At the macroscopic scale, tissue is treated as a porous medium in LTE, governed by a single temperature field for both solid and fluid phases. The classical Pennes’ bioheat equation is expressed as [31,44]:

(3) $$\rho c\frac{\partial T}{\partial t}=\nabla \cdot \left(k\nabla T\right)+Q_{\mathrm{RF}}+Q_{\mathrm{perf}},$$

where ρ, *c*, and *k* denote effective density, heat capacity, and thermal conductivity, respectively, and $Q_{\mathrm{RF}}$ represents Joule heating, while $Q_{\mathrm{perf}}$ denotes the perfusion-induced heat transfer. Conventional formulations approximate perfusion via isotropic volumetric source terms, inherently neglecting directional advective heat transport driven by microvascular blood flow. Accordingly, the present framework adopts a modified LTE formulation that incorporates an explicit advective term derived from volume-averaged blood velocity:

(4) $$\left[\left(1-\varepsilon_{\mathrm{var}}\right){\left(\rho c\right)}_{t}+\varepsilon_{\mathrm{var}}{\left(\rho c\right)}_{b}\right]\frac{\partial T}{\partial t}+\varepsilon_{\mathrm{var}}{\left(\rho c\right)}_{b}\beta S=\left[\left(1-\varepsilon_{\mathrm{var}}\right)k_{t}+\varepsilon_{\mathrm{var}}k_{b}\right]{▿}^{2}T+Q_{\mathrm{RF}},$$

(5) $${\left(\rho c\right)}_{t}=\left\{\begin{array}{cc} {\left(\rho_{l}c_{l}\right)}_{t}, & 0\;{}^{\circ}C<T\leq 99\;{}^{\circ}C\\ \frac{h_{fg}C_{w,t}}{\Delta T_{b,t}}, & 99\;{}^{\circ}C<T\leq 100\;{}^{\circ}C\\ \rho_{g}c_{g}, & T>100\;{}^{\circ}C \end{array}\right.$$

(6) $${\left(\rho c\right)}_{b}=\left\{\begin{array}{cc} {\left(\rho_{l}c_{l}\right)}_{b}, & 0\;{}^{\circ}C<T\leq 99\;{}^{\circ}C\\ \frac{h_{fg}C_{w,b}}{1\;{}^{\circ}C}, & 99\;{}^{\circ}C<T\leq 100\;{}^{\circ}C\\ \rho_{g}c_{g}, & T>100\;{}^{\circ}C \end{array}\right.$$

(7) $$k\left(T\right)=\left\{\begin{array}{cc} k_{0}+\Delta k\left(T-T_{0}\right), & T\leq 100\;{}^{\circ}C\\ k_{0}+\Delta k\left(100\;{}^{\circ}C-T_{0}\right), & T>100\;{}^{\circ}C \end{array}\right.$$

with Δk=0.0013. Here, $T_{0}=$ 37 °C is the reference temperature. The piecewise definitions for ρ(T) and c(T) reflect the thermophysical shifts due to vaporization and tissue desiccation [42]. β is the coefficient which is 0 for $\theta_{d}\geq 0.99$, and 1 in other cases.

For liver tissue and blood, density ρ and specific heat capacity $c_{p}$ exhibit discontinuous changes above 100 °C to account for interstitial water vaporization and tissue desiccation [42]. Thermal conductivity *k* and the reference electrical conductivity $\sigma_{0}$ are treated as temperature-independent in the baseline model, while the strong nonlinear dependence of electrical conductivity is captured by the multiplicative scaling factor in Equation (2). Tumor tissue properties are assumed temperature-invariant across the simulated range, consistent with prior modeling frameworks [42]. All adopted thermophysical and electrical material properties used in the numerical simulations are summarized in Table 1.

**Table 1:** Thermophysical and electrical properties of materials used in simulations. Parameter values are taken from [42]. Temperature-dependent properties for liver tissue and blood are specified for regimes below and above 100 °C.

Material	Regime	ρ (kg/m^3^)	*c* (J/(kg · K))	$k_{0}$ (W/(m · K))	$\sigma_{0}$ (S/m)
Liver Tissue	T< 100 °C	1080 *	3455 *	0.515	0.203 ***
Liver Tissue	T≥ 100 °C	370 **	2156 **	0.515	0.203 ***
Liver tumor	–	1045	3760	0.6	0.5
Blood	T< 100 °C	1000 *	3639 *	0.49	0.667
Blood	T≥ 100 °C	370 **	2156 **	0.49	0.667
Electrode	–	8000	480	15	$7.4\times 10^{6}$
Plastic (insulator)	–	70	1045	0.026	$10^{-5}$

* Pre-ablation, ** post-vaporization, and *** reference at 37 °C.

In contrast to conventional approaches where the advective term is explicitly prescribed, the scalar product $S=v_{\mathrm{eff}}\cdot \nabla T$ is computed via an auxiliary finite-element equation solved concurrently with the thermal field. This ensures numerical consistency between the discretized temperature gradient and advective transport, eliminating phenomenological averaging at the element level. The effective velocity $v_{\mathrm{eff}}$ is oriented radially from the electrode, capturing volume-averaged perfusion without resolving discrete vasculature, while $\rho_{b}$ and $c_{b}$ denote blood density and specific heat capacity. Coupled with dynamic property updates driven by temperature and accumulated damage, this formulation establishes a tightly coupled feedback loop between heat deposition, tissue necrosis, and electrical conductivity.

### 2.3. Spatial Porosity Distribution

**Figure 2:**
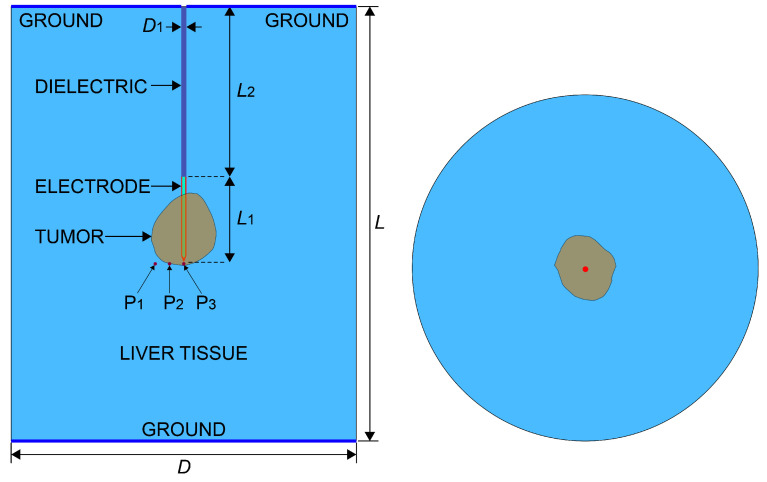
Schematic of the computational domain showing the RFA electrode inserted into a patient-specific tumor geometry. Control points P1–P3 denote locations for monitoring local temperature, electric potential, and thermal tissue damage.

**Figure 3:**
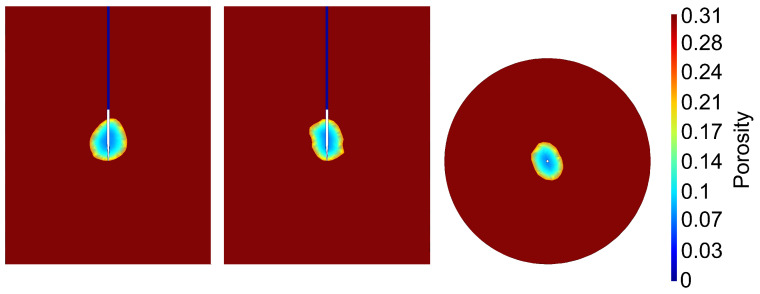
Spatial distribution of tissue porosity across the tumor volume. The geometry-informed field definition directly incorporates microstructural heterogeneity into the multiphysics computa-tional domain.

The patient-specific tumor geometry defines the computational domain, as illustrated in Figure 2. Within this domain, tissue porosity is implemented as a spatially varying scalar field assigned to each finite element via the Gmsh meshing framework. Instead of imposing idealized radial profiles, this geometry-informed approach captures continuous porosity gradients across the tumor volume, transitioning from low-porosity central regions ($\varepsilon_{\mathrm{var}}=0.07$) to highly vascularized peripheral tumor ($\varepsilon_{\mathrm{var}}=0.23$), as visualized in Figure 3. By mapping porosity directly onto the computational mesh, structural heterogeneity is preserved without geometric simplifications, allowing electromagnetic field distributions and advective heat transport to be directly governed by the local tissue architecture.

## 3. Results

**Figure 4:**
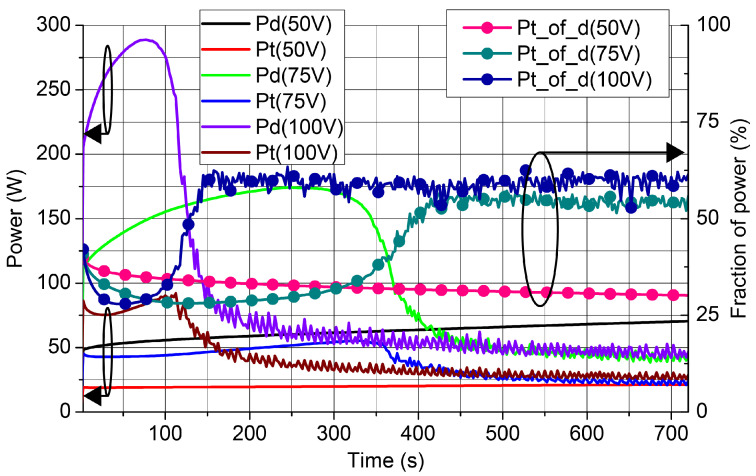
Distribution of absorbed power in the liver computational domain and tumor at input voltages of 50 V, 75 V, and 100 V, with tumor power fraction (percentage of total power delivered to tumor mass).

Total absorbed power exhibited strong voltage-dependent nonlinearity, as can be observed from Figure 4. At 100 V, power peaked at ≈280 W (75 s) before declining to 40–80 W, reflecting initial conductivity enhancement followed by dehydration-induced collapse. The tumor power fraction increased from ≈30% to ≈60%, indicating progressive current confinement as surrounding tissue impedance rose. At 75 V, peak power (≈175 W) occurred later (300 s) with smoother transition to steady state. At 50 V, power remained low (50–70 W) with minimal conductivity changes, yielding weak energy focusing. Higher relative tumor power reflects spatial confinement rather than improved efficacy; effective ablation requires balancing total power delivery with controlled localization.

**Figure 5:**
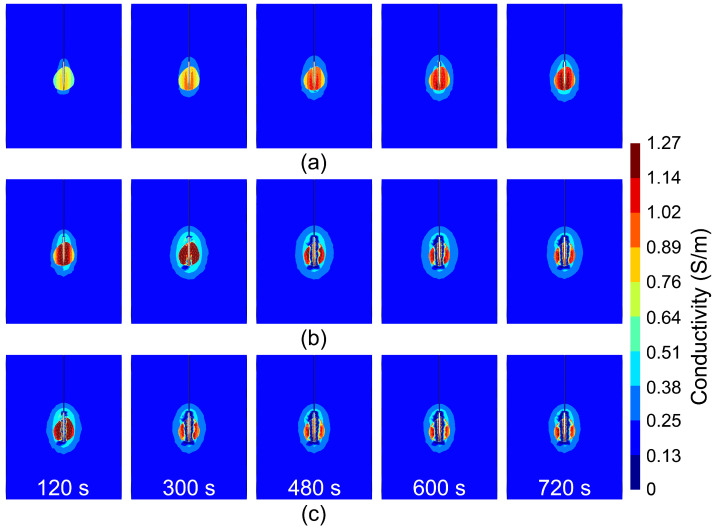
Numerically predicted temporal evolution of electrical conductivity distribution at input voltages of (**a**) 50 V, (**b**) 75 V, and (**c**) 100 V.

Electrical conductivity evolution revealed three distinct electro-thermal regimes (Figure 5). At 50 V, conductivity increased monotonically throughout the simulation (720 s), remaining spatially confined to the tumor without evidence of conductivity collapse (Figure 5a). At 75 V, localized conductivity reduction emerged near the electrode at ≈300 s and progressed to dominate the 480–720 s interval as dehydration established a low-conductivity peri-electrode layer (Figure 5b). At 100 V, conductivity collapse initiated by 120 s and was fully developed by 300 s, accounting for the early power peak and subsequent rapid decay (Figure 5c). These results demonstrate a voltage-dependent transition from thermally enhanced conductivity (low voltage) to damage-driven conductivity collapse (high voltage), mediated by dehydration and coagulative necrosis.

**Figure 6:**
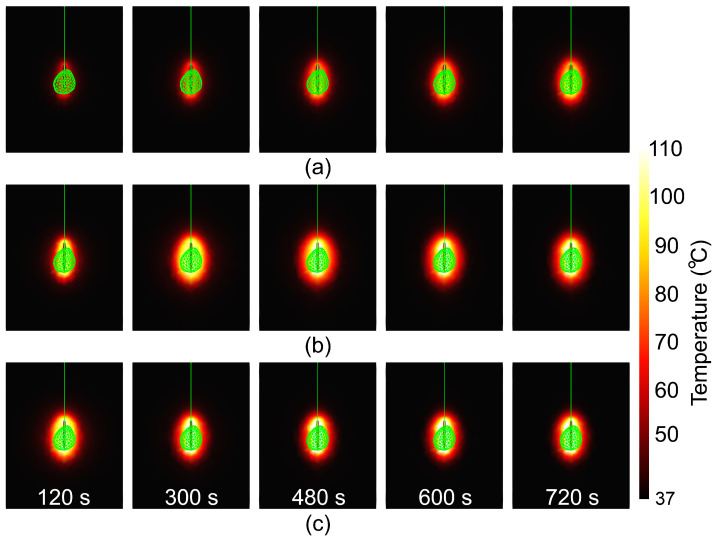
Spatiotemporal temperature distributions predicted numerically at input voltages of (**a**) 50 V, (**b**) 75 V, and (**c**) 100 V. Green curves represent tumor.

Figure 6 presents spatiotemporal temperature distributions for input voltages of (a) 50 V, (b) 75 V, and (c) 100 V. At 50 V (Figure 6a), temperature rises gradually, reaching ≈100 °C only after 720 s; this moderate heating preserves electrical coupling and sustains steady radial heat propagation. At 75 V (Figure 6b), temperature surpasses 100 °C within 120 s and achieves optimal spatial uniformity by ≈300 s. At 100 V (Figure 6c), intense initial heating induces a rapid drop in electrical conductivity near the electrode, limiting further energy transfer despite elevated local temperatures.

**Figure 7:**
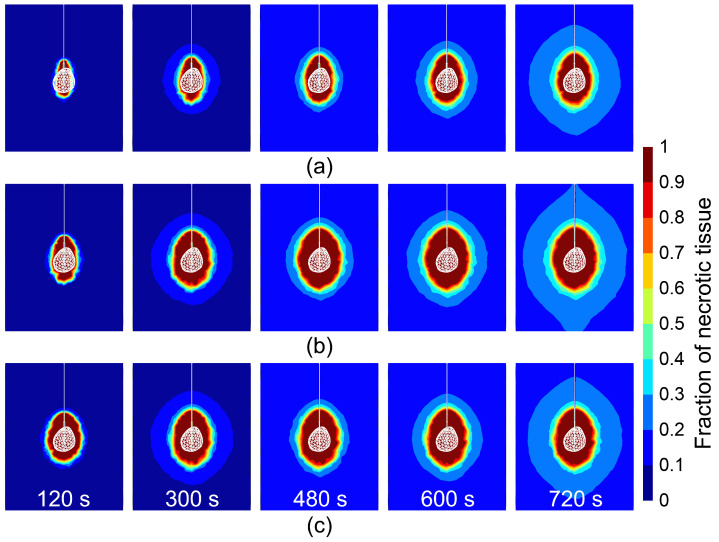
Spatiotemporal evolution of the tissue damage fraction (necrosis) at input voltages of (**a**) 50 V, (**b**) 75 V, and (**c**) 100 V, computed using the Arrhenius kinetic model with a damage threshold of Ω=4.6 ($\theta_{d}\geq 0.99$).

Figure 7 shows the evolution of the tissue damage fraction corresponding to the temperature fields in Figure 6. At 50 V (Figure 7a), necrosis develops gradually, ultimately encompassing the tumor with moderate safety margins. At 75 V (Figure 7b), complete tumor necrosis is achieved ≈2× faster than at 50 V, though damage expansion decelerates after 300 s owing to reduced electrical conductivity near the electrode. At 100 V (Figure 7c), necrosis stabilizes after 300 s at a spatial extent comparable to the 75 V case, with negligible subsequent progression.

**Figure 8:**
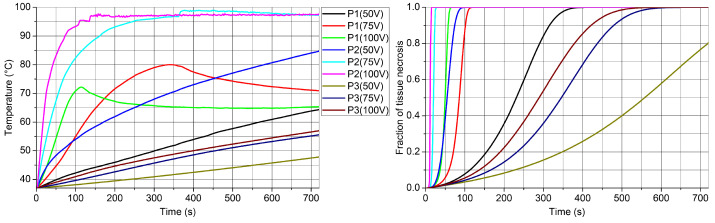
Time-dependent temperature and tissue damage fraction at control points P1–P3 for input voltages of 50 V, 75 V, and 100 V.

Figure 8 illustrates the temperature and necrosis dynamics at three control points: P3 (electrode tip), P2 (5 mm lateral), and P1 (10 mm lateral). At P3, high voltages induce a rapid temperature rise but delay necrosis onset owing to early dehydration near the electrode. At P2, complete tissue damage is achieved within 15 s at 100 V and 28 s at 75 V, identifying this region as the zone of optimal energy transfer. At P1, temperature progression decelerates at higher voltages, reflecting current redistribution driven by conductivity gradients. Collectively, these results demonstrate that ablation efficacy is governed by spatially distributed electro-thermal feedback rather than peak temperature near the electrode alone.

## 4. Discussion

This study demonstrates that dynamic electrical conductivity evolution, driven by spatially varying tissue porosity and thermal damage, governs energy deposition and ablation efficacy in radiofrequency ablation (RFA). By coupling patient-specific 3D geometry with a modified local thermal equilibrium formulation that explicitly resolves advective heat transport, the framework captures electro-thermal feedback mechanisms absent in conventional homogeneous models. The simulated temporal heating profiles, peri-electrode temperatures (100–105 °C at 75 V), and radial thermal gradients (≈15 °C mm^−1^) closely match experimental miniature-sensor measurements [15]. Furthermore, predicted necrotic diameters (2.8 ± 0.4 cm at 720 s) align with MRI-validated clinical benchmarks [21], while the reproduced 60–80% conductivity decline accurately reflects the impedance rise signaling peri-electrode desiccation and self-limiting energy transfer.

While clinical RFA protocols employ a continuous spectrum of voltage settings, the three representative levels examined here (50, 75, and 100 V) were deliberately selected to delineate the key electro-thermal regimes governing ablation dynamics: (i) stable conductivity and gradual thermal propagation at low voltage, (ii) optimal energy coupling with controlled tissue desiccation at intermediate voltage, and (iii) premature conductivity collapse that confines current to peripheral viable tissue at high voltage. The underlying governing equations vary continuously with applied potential, indicating that intermediate settings (e.g., 60–90 V) exhibit smoothly interpolating behavior. This expectation is supported by preliminary simulations at 70, 80, and 90 V, which confirmed monotonic transitions in power deposition, conductivity evolution, and necrosis progression without introducing qualitatively new regimes. Consequently, while exhaustive parameter sweeps may refine optimal voltage boundaries for specific anatomical contexts, they are not expected to alter the fundamental conclusion that ablation efficacy is governed by spatially distributed electro-thermal feedback rather than peak applied voltage alone.

From a clinical perspective, these findings have direct implications for intraoperative protocol selection and monitoring. The early conductivity collapse observed at 100 V illustrates why aggressive power settings may paradoxically limit lesion growth by inducing rapid peri-electrode desiccation—a phenomenon clinically recognized as impedance roll-off. Conversely, the sustained coupling at 75 V supports moderate, prolonged energy delivery to achieve complete tumor coverage while preserving current pathways. This reinforces the value of impedance trend analysis, rather than absolute thresholds, as a real-time diagnostic indicator of ablation progression. Furthermore, the geometry-informed porosity mapping employed here is explicitly designed for future integration with contrast-enhanced CT or multiparametric MRI. Once derived from radiomic texture analysis or machine-learning segmentation, patient-specific porosity fields could enable pre-procedural simulation of impedance trajectories, allowing tailored voltage titration that accounts for individual tumor histology and adjacent vasculature.

The robustness of the identified regimes was evaluated through localized sensitivity analysis, varying Arrhenius constants, porosity gradients, and effective perfusion velocity independently by ±20%. Across all perturbations, the qualitative hierarchy of operating regimes remained unchanged, and the ablation efficacy ranking (75 V > 50 V > 100 V) was preserved, with only absolute necrosis times exhibiting moderate variability (±15%). This confirms that the observed electro-thermal transitions are inherent structural features of the coupled system rather than artifacts of specific parameter choices. Nevertheless, several limitations warrant acknowledgment. The assumption of radially symmetric effective perfusion, while computationally efficient, does not fully capture patient-specific vascular architectures. Additionally, validation against aggregated literature benchmarks cannot substitute for prospective patient-matched imaging correlation, and the piecewise approximations for temperature-dependent properties may not capture the full continuum of tissue transformation during ablation. The current baseline also assumes non-cirrhotic parenchyma; however, the porosity field can be calibrated to reflect fibrotic or steatotic conditions by adjusting baseline transport parameters.

Regarding clinical translation, the geometry-informed porosity mapping employed here is explicitly designed for future integration with imaging-derived patient-specific fields. In practice, spatial porosity distributions can be approximated from contrast-enhanced CT or multiparametric MRI using radiomic texture analysis or machine-learning-based segmentation of vascular and extracellular matrix patterns. Once integrated, these maps would enable pre-procedural simulation of patient-specific impedance trajectories, allowing tailored voltage and duration protocols that account for underlying parenchymal disease (e.g., fibrosis, steatosis) and tumor histology. This pathway positions the model as a decision-support tool for personalized RFA planning, reducing empirical guesswork and improving procedural safety.

## 5. Conclusions

This work establishes spatially varying tissue porosity as a critical determinant of electro-thermal feedback in radiofrequency ablation. By resolving the coupling between microstructural heterogeneity and dynamic conductivity evolution, the proposed framework addresses a fundamental limitation of conventional homogeneous models. The key advance lies not merely in improved geometric fidelity, but in enabling mechanistic interpretation of intraoperative impedance dynamics as a window into the underlying tissue state.

Several limitations warrant acknowledgment. The assumption of radially symmetric effective perfusion, while computationally efficient, does not capture patient-specific vascular architectures that may influence heat dissipation patterns. Additionally, validation against aggregated literature benchmarks, while demonstrating consistency with established ranges, cannot substitute for prospective patient-matched imaging correlation. The temperature-dependent property updates, though physically motivated, rely on piecewise approximations that may not fully capture the continuum of tissue transformation during ablation.

Future research directions should prioritize three areas: First, integration of contrast-enhanced pre-procedural imaging to derive patient-specific porosity maps rather than geometry-informed estimates; second, global sensitivity analysis to quantify the relative influence of Arrhenius parameters, porosity gradients, and perfusion assumptions on predicted ablation margins; and third, prospective clinical validation comparing model-predicted impedance trajectories against intraoperative measurements and post-ablation MRI confirmation.

## Data Availability

The raw data supporting the conclusions of this article will be made available by the authors on request.
